# Antimicrobial Resistance and Type III Secretion System Virulotypes of Pseudomonas aeruginosa Isolates from Dogs and Cats in Primary Veterinary Hospitals in Japan: Identification of the International High-Risk Clone Sequence Type 235

**DOI:** 10.1128/Spectrum.00408-21

**Published:** 2021-09-29

**Authors:** Wataru Hayashi, Katsutoshi Izumi, Satoshi Yoshida, Shino Takizawa, Kanae Sakaguchi, Keita Iyori, Ken-ichi Minoshima, Shinya Takano, Maki Kitagawa, Yukiko Nagano, Noriyuki Nagano

**Affiliations:** a Department of Medical Sciences, Shinshu University Graduate School of Medicine, Science and Technology, Nagano, Japan; b Department of Health and Medical Sciences, Shinshu University Graduate School of Medicine, Nagano, Japan; c VDT Co., Ltd., Tokyo, Japan; d Research and Development Division, Kyokuto Pharmaceutical Industrial Co., Ltd., Tokyo, Japan; e Nagoya University Graduate School of Medicine, Nagoya, Japan; University of Manitoba

**Keywords:** *Pseudomonas aeruginosa*, companion animal, T3SS, ST235

## Abstract

This study aimed to investigate the current trends in antimicrobial resistance among Pseudomonas aeruginosa clinical isolates of canine and feline origin and the prevalence of their sequence types (STs) and type III secretion system (T3SS) virulotypes, which remains unknown in Japan. A total of 240 nonduplicate clinical isolates of P. aeruginosa from dogs (*n *= 206) and cats (*n *= 34) collected from 152 primary care animal hospitals between August 2017 and October 2019 were examined. PCR detection of T3SS genes (*exoU* and *exoS*) and carbapenemase genes, multilocus sequence typing, and whole-genome sequencing of the representative carbapenem-resistant isolates were performed. Resistance rates to imipenem and meropenem were 6.67% and 2.08%, respectively. A high resistance rate (17.92%) was encountered with ciprofloxacin. The *exoU*−/*exoS*+ was the predominant T3SS virulotype (195 isolates, 81.3%), followed by *exoU*+/*exoS*− (35 isolates, 14.6%), *exoU*−/*exoS*− (7 isolates, 2.9%), and *exoU*+/*exoS*+ (3 isolates, 1.3%). A high frequency of the high-risk clones ST235 and clonal complex 235 (CC 235) (28.9%), followed by ST357 (21.1%), were noted among these 38 *exoU*+ isolates. Seventeen carbapenem-resistant isolates comprising 2 *exoU*+ isolates, including an ST235 isolate, and 15 *exoU*−/*exoS*+ isolates belonging to non-ST235/CC235 were detected, of which all were carbapenemase negative. Different combinations of mutations among *oprD*, efflux pump regulatory genes, and AmpC β-lactamase regulatory genes were identified among representative isolates with high-level resistance to imipenem. This study emphasizes the occurrence of ST235 isolates among companion animals, which may represent a threat to public health because of the ability of this clone to acquire and spread resistance elements, including carbapenemase genes.

**IMPORTANCE** Pseudomonas aeruginosa is an environmentally ubiquitous and important opportunistic human pathogen responsible for life-threatening health care-associated infections. Because of its extensive repertoire of virulence determinants and intrinsic and acquired resistance mechanisms, the organism could be one of the most clinically and epidemiologically important causes of morbidity and mortality. In recent years, worldwide spreading of multidrug-resistant high-risk clones, particularly sequence type 235 (ST235), has become a serious public health threat. Companion animals which share much of their living environment with humans could be important reservoirs and spreaders of antimicrobial-resistant bacteria and resistance genes of clinical importance in humans, such as extended-spectrum β-lactamase-producing *Enterobacterales* and methicillin-resistant Staphylococcus aureus. However, antimicrobial resistance, virulence, and genotyping of P. aeruginosa in companion animals remain largely unknown. This work sheds light on the potential spread of high-risk clones in companion animals.

## INTRODUCTION

Pseudomonas aeruginosa is an important opportunistic human pathogen capable of causing a wide variety of life-threatening acute and chronic infections and is also a major cause of health care-associated infections. The bacterium is environmentally ubiquitous, inhabiting soil, water, plants, and animals, and can be isolated from the skin, throat, and stool of healthy persons. P. aeruginosa is equipped with an extensive repertoire of virulence determinants implicated in pathogenesis and intrinsic and acquired resistance mechanisms, which makes the organism one of the most clinically and epidemiologically important causes of morbidity and mortality (1).

Recent studies have raised attention to the increasing worldwide prevalence of P. aeruginosa high-risk clones that are multidrug resistant (MDR) or extensively drug resistant (XDR) in hospitals. Those internationally recognized high-risk clones include sequence type 235 (ST235), ST111, ST233, ST244, ST357, ST308, ST175, ST277, ST654, and ST298 (2). Carbapenem resistance in P. aeruginosa can be mediated by several mechanisms, including the interaction of OprD inactivation, Mex efflux system overexpression, and intrinsic AmpC β-lactamase overexpression and the production of acquired carbapenemases. VIM- and IMP-type metallo-β-lactamases are the most commonly encountered carbapenemases in P. aeruginosa, including international high-risk clones, although other carbapenemases, such as KPC, GES, NDM, and SPM, have also been reported globally (2). In Japan, GES, IMP, and VIM types have so far been identified among carbapenemase-producing high-risk clones, including ST235, ST244, ST357, ST308, ST175, and ST277 (2). Particularly, outbreaks of ST235 producing IMP type or GES 5 have been reported in clinical settings in Japan (3, 4).

One of the most important virulence factors resulting in a poor prognosis of infections is the type III secretion system (T3SS) (5). The system injects bacterial effector exotoxins, including ExoU (phospholipase A2), ExoS (Rho GTPase-activating protein [RhoGAP]/ADP-ribosyltransferase [ADPRT]), ExoT (RhoGAP/ADPRT), and ExoY (adenyl cyclase) identified so far, into host cells (6). ExoU and ExoS are responsible for the cytotoxic phenotype and invasive phenotype, respectively, contributing more significantly to pathogenesis than the other exotoxins (7, 8). ExoU is located in pathogenicity island 2 (PAPI-2), and ExoS shows mostly mutually exclusive distributions, while ExoT and ExoY are carried by most of the strains (9). The *exoU*+ genotype has been frequently associated with multidrug resistance, fluoroquinolone resistance, and highly virulent phenotypes, as represented by the ST235 high-risk clone, resulting in increased mortality in bloodstream infections (10–12).

Companion animals have been recognized as potential reservoirs of antimicrobial resistance genes and resistant bacteria that can be transmitted to humans (13, 14). However, studies on P. aeruginosa virulence and antimicrobial resistance in companion animals are scarce and have focused mostly on human isolates. In France, ST233 and potential high-risk clones ST395 and ST253 have been found among 29 carbapenemase-negative carbapenem-nonsusceptible P. aeruginosa clinical isolates of canine and feline origin (15). VIM-2-positive ST233 and IMP-45-positive ST308 P. aeruginosa have been reported to be associated with possible human-to-dog transmission in Brazil and China (16, 17). In a US canine study, *exoU−*/*exoS*+, *exoU*+/*exoS−*, and *exoU*-/*exoS−* T3SS virulotypes have been identified in 15, 3, and 1 isolates, respectively, among 19 isolates from ocular infections, while *exoU−*/*exoS*+ and *exoU*+/*exoS*+ virulotypes have been detected in 5 and 1 isolates, respectively, among 6 normal conjunctival microflora isolates (18).

In Japan, only a few epidemiological studies are available to date on the antimicrobial resistance of P. aeruginosa clinical isolates of companion animal origin (19, 20). A recent study has documented the low rates of resistance to ceftazidime (0.5%), ciprofloxacin (9%), and amikacin (2.5%), and neither imipenem-resistant isolates nor metallo-β-lactamase producers have been identified (20). This study investigates the current trends in antimicrobial resistance among P. aeruginosa clinical isolates of canine and feline origin and the prevalence of their STs and T3SS virulotypes, which remains unknown in Japan, to better understand their epidemiological aspects.

## RESULTS

### Prevalence of antibiotic resistance and T3SS virulotypes among P. aeruginosa isolates.

The ranges of MICs for 240 isolates of P. aeruginosa against imipenem and meropenem were ≤1 to >64 mg/liter and ≤0.5 to 16 mg/liter, respectively. The rates of resistance were 6.67% (16 isolates) for imipenem and 2.08% (5 isolates) for meropenem (Table 1). Notably, those results are comparable to the corresponding carbapenem resistance rates, namely, 5.7% for imipenem and 2.8% for meropenem in P. aeruginosa isolates among outpatients in 2019 obtained from the Japan Nosocomial Infections Surveillance (JANIS) database (Table 1) (https://janis.mhlw.go.jp/report/open_report/2019/3/1/ken_Open_Report_201900_Outpatient.pdf). In contrast, the resistance rates against piperacillin, piperacillin-tazobactam, ceftazidime, and cefepime were 0.83% (2 isolates), 0.83% (2 isolates), 0.83% (2 isolates), and 0.42%, (1 isolate), respectively, which were lower than those from JANIS data of outpatient isolates. The resistance levels of aminoglycosides were 0.42% (1 isolate) for amikacin, 2.08% (5 isolates) for gentamicin, and 1.67% (4 isolates) for tobramycin. A high resistance rate was encountered with ciprofloxacin (43 isolates, 17.92%), surpassing the resistance rate against levofloxacin among clinical isolates from both inpatients (9.8%) and outpatients (6.0%). Individual MIC values for each isolate are listed in Table S1 in the supplemental material.

**TABLE 1:** The MICs and the antimicrobial resistance rates for the 240 P. aeruginosa isolates from dogs and cats

Antimicrobial agent(s)	MIC range (mg/liter)	MIC_50_ (mg/liter)	MIC_90_ (mg/liter)	Resistance (%)	2019 JANIS data^*a*^ on human clinical isolates (resistance %) from:	
					Inpatients	Outpatients
Piperacillin	≤4 to >128	8	16	0.83	10.3	3.4
Piperacillin-tazobactam	≤8/4 to 128/4	≤8/4	16/4	0.83	8.4	2.5
Ceftazidime	≤2 to 64	≤2	8	0.83	8.7	2.9
Cefepime	≤2 to 32	4	8	0.42	5.9	2.3
Aztreonam	≤2 to 64	8	32	11.25	13.3	6.5
Imipenem	≤1 to >64	2	4	6.67	16.2	5.7
Meropenem	≤0.5 to 16	≤0.5	2	2.08	10.6	2.8
Gentamicin	≤1 to >32	2	4	2.08	3.1	2.6
Amikacin	≤2 to >64	4	8	0.42	0.9	0.8
Tobramycin	≤2 to >32	≤2	≤2	1.67	NA^*b*^	NA
Ciprofloxacin	≤0.5 to >8	≤0.5	4	17.92	9.8 (levofloxacin)	6.0 (levofloxacin)

a JANIS, Japan Nosocomial Infections Surveillance.

b NA, not available.

The presence of 40 mg/liter phenylalanine-arginine β-naphthylamide (PaβN) did not inhibit the growth of 43 ciprofloxacin-resistant P. aeruginosa isolates except 1, for which the PAβN concentration not affecting its growth (20 mg/liter) was used for the efflux pump inhibition. All 43 isolates, including 7 carbapenem-resistant isolates, showed more than or equal to a 4-fold reduction in the MIC of ciprofloxacin in the presence of PAβN (data not shown).

Overall, the frequency of *exoU* and *exoS* genes was 15.8% (38/240 isolates) and 82.5% (198/240 isolates), respectively. PCR revealed 4 T3SS virulotypes among 240 isolates, namely, 35 *exoU*+/*exoS*− isolates (14.6%), 195 *exoU*−/*exoS*+ isolates (81.3%), 3 *exoU*+/*exoS*+ isolates (1.3%), and 7 *exoU*−/*exoS*− isolates (2.9%). Table 2 shows the distribution of T3SS virulotypes according to antimicrobial resistance. The *exoU*+/*exoS*− isolates, compared with the *exoU*−/*exoS*+ isolates, were significantly associated with resistance to piperacillin (*P = *0.02843), piperacillin-tazobactam (*P = *0.03787), gentamicin (*P = *0.009844), and tobramycin (*P = *0.04685). Alternatively, no statistically significant association was found between these virulotypes and resistance to imipenem, meropenem, and ciprofloxacin.

**TABLE 2:** Distribution of T3SS virulotypes among P. aeruginosa clinical isolates from companion animals according to antimicrobial resistance

Antimicrobial(s)	No. (%) of isolates by T3SS virulotype				*P* value for comparison between *exoU*+/*exoS*− and *exoU*−/*exoS*+
	*exoU*+/*exoS*− (*n* = 35)	*exoU*−/*exoS*+ (*n* = 195)	*exoU*+/*exoS*+ (*n* = 3)	*exoU*−/*exoS*− (*n* = 7)	
Piperacillin	6 (17)	11 (6)	0	0	0.02843
Piperacillin-tazobactam	6 (17)	12 (6)	0	0	0.03787
Ceftazidime	1 (3)	5 (3)	0	0	
Cefepime	1 (3)	12 (6)	0	0	
Aztreonam	8 (23)	49 (25)	0	0	
Imipenem	4 (11)	41 (21)	0	0	
Meropenem	2 (6)	11 (6)	0	0	
Gentamicin	6 (17)	8 (4)	0	0	0.009844
Amikacin	0 (0)	2 (1)	0	0	
Tobramycin	3 (9)	3 (2)	0	0	0.04685
Ciprofloxacin	6 (17)	60 (31)	0	1	
Colistin	0 (0)	1 (1)	0	0	

### Multilocus sequence typing (MLST) of *exoU*+ isolates.

The 35 *exoU*+/*exoS*− isolates were assigned to 20 STs from which 2 new STs, namely, ST235-like and ST3654, were identified (Table 3). The 3 *exoU*+/*exoS*+ isolates belonged to different STs, including a new ST, ST3653. The high frequency of high-risk clone ST235 (8, 21.1%) and ST141 (1, 2.6%), ST235-like (1, 2.6%), and ST3653 (1, 2.6%) belonging to CC235 was noted among those 38 *exoU*+ isolates (11, 28.9%). Other high-risk clones and clonal complexes identified were ST357 (8, 21.1%), ST2644 (CC274) (3, 7.9%), ST308 (2, 5.3%), ST253 (1, 2.6%), and ST446 (CC298) (1, 2.6%).

**TABLE 3:** STs identified among the 38 *exoU*+ P. aeruginosa clinical isolates from companion animals

MLST sequence type	Clonal complex	No. of clinical isolates by virulotype and animal				Total no. of isolates
		*exoU*+/*exoS*− (*n* = 35)		*exoU*+/*exoS*+ (*n* = 3)		
		Dog	Cat	Dog	Cat	
ST235	CC235	5	2	1		8
ST141		1				1
ST235-like^*a*^		1				1
ST3653^*b*^				1		1
ST357	CC357	7		1		8
ST2644	CC274	3				3
ST308	CC308	2				2
ST671	CC560	1	1			2
ST253	CC253	1				1
ST313	CC313	1				1
ST316	CC316	1				1
ST2555		1				1
ST446	CC298	1				1
ST606	NA^*c*^	1				1
ST773	NA	1				1
ST829	NA	1				1
ST1121	NA	1				1
ST1248	NA	1				1
ST1334	NA	1				1
ST3654^*b*^	NA	1				1
Total		32	3	3		38

a Novel *trpE* allele was associated with ST235-like.

b Sequence types newly assigned in this study.

c NA, not assigned.

### Phenotypic characteristics of carbapenem-resistant P. aeruginosa isolates.

Among the 240 isolates, 17 (7.1%) were resistant to imipenem and/or meropenem. Detection of carbapenemase production by a modified carbapenem inactivation method using Tris-HCl (CIMTris) assay yielded negative results for all 17 isolates. In those isolates, the major carbapenemase genes *bla*_IMP_, *bla*_NDM_, *bla*_VIM_, and *bla*_GES_ were not detected (Table 4).

**TABLE 4:** Features of the 17 carbapenem-resistant P. aeruginosa isolates

Isolate		Animal species	Clinical sample	MLST sequence type	T3SS virulotype		Carbapenemase detection		MIC (mg/liter)^*a*^ of:							
No.	Name				*exoU*+/*exoS*−	*exoU*−/*exoS*+	CIMTris	PCR detection of *bla*_IMP_, *bla*_NDM_, *bla*_VIM_, and *bla*_GES_	IPM	IPM + PAβN	IPM + APB	IPM + PaβN + APB	MEPM	MEPM + PAβN	MEPM + APB	MEPM + PaβN + APB
1	CA10562	Dog	Ear discharge	ST3014 (CC3014)	−	+	−	−	8	**1**	**0.5**	**≤0.25**				
2	CA12133	Dog	Ear discharge	ST1600	−	+	−	−	64	**4**	**8**	**1**				
3	CA12482	Cat	Skin	ST198 (CC198)	−	+	−	−	16	16	**1**	**1**				
4	CA13227	Dog	Ear discharge	ST209 (CC274)	−	+	−	−	8	8	**1**	**1**				
5	CA13620	Dog	Ear discharge	ST277 (CC277)	−	+	−	−	4	2	**0.5**	**≤0.25**	4	4	4	2
6	CA13876	Cat	Nasal discharge	ST1097	−	+	−	−	8	4	**0.5**	**≤0.25**				
7	CA14241	Dog	Ear discharge	ST3045	−	+	−	−	16	16	**1**	**2**				
8	CA16138	Dog	Ear discharge	ST3574^*b*^ (CC262)	−	+	−	−	8	**2** ^*c*^	4	**1** ^*c*^				
9	CA16209	Dog	Ear discharge	ST606	+	−	−	−	8	**1**	**0.5**	**≤0.25**				
10	CA17343	Dog	Uterine pus	ST348 (CC348)	−	+	−	−	128	**8** ^*c*^	**4**	**0.5** ^*c*^	16	**2** ^*c*^	8	**2** ^*c*^
11	CA17462	Dog	Urine	ST270	−	+	−	−	4	**1**	**0.5**	**≤0.25**				
12	CA17502	Dog	Ear discharge	ST155 (CC155)	−	+	−	−					16	**4**	8	**4**
13	CA19603	Cat	Ear discharge	ST235 (CC235)	+	−	−	−	32	16	**2**	NG^*d*^	8	**1**	4	NG^*d*^
14	CA19802	Dog	Urine	ST399 (CC399)	−	+	−	−	32	16	**2**	**2**	16	8	8	**4**
15	CA19818	Dog	Urine	ST266	−	+	−	−	4	2	**≤0.25**	**≤0.25**				
16	CA20091	Dog	Ear discharge	ST348 (CC348)	−	+	−	−	4	4	**0.5**	**0.5**				
17	CA20115	Dog	Urine	ST3135	−	+	−	−	4	8	**0.5**	**0.5**				

a A decrease of more than 4-fold in MIC values are indicated in bold.

b Sequence type newly assigned in this study.

c PAβN concentration of 20 mg/liter was used.

d No visible growth was observed in the presence of imipenem or meropenem plus 20- or 40-mg/liter PAβN and 300-mg/liter APB.

The presence of 40-mg/liter PAβN did not inhibit the growth of 17 carbapenem-resistant P. aeruginosa, except for 2 isolates (no. 8 and 10), for which PAβN concentration not affecting their growth (20 mg/liter) was used for the efflux pump inhibition. A 4-fold or greater decrease in the MICs of imipenem and/or meropenem with the addition of PAβN was observed in 8 of 17 isolates (47.1%) (Table 4). In the presence of 3-aminophenylboronic acid (APB), an inhibitor of AmpC β-lactamase, most isolates (15/16, 93.8%) exhibited a greater than or equal to an 8-fold reduction in the MICs of imipenem, while MICs of meropenem for 5 isolates were not affected. It was noticed that APB and PAβN plus APB effectively change interpretive categories from imipenem resistant/intermediate (MIC, ≥8/4 mg/liter) to susceptible (MIC, ≤2 mg/liter) in 13 (81.3%) and 15 isolates (93.8%), respectively. Meropenem MIC of an isolate (no. 12) with the imipenem-susceptible, meropenem-resistant phenotype was affected by PAβN (4-fold reduction) but was not affected by APB.

### Genotypic characteristics of carbapenem-resistant P. aeruginosa isolates.

MLST sequence types and T3SS virulotypes of 17 carbapenem-resistant isolates are shown in Table 4. These isolates were assigned to 16 STs, including a newly identified ST3574 (CC262). They contained 2 *exoU*+/*exoS*− isolates and 15 *exoU*−/*exoS*+ isolates. It was of note that high-risk international clones ST235 with an *exoU*+/*exoS*− virulotype (no. 13) and ST277 with an *exoU*-/*exoS*+ virulotype (no. 5) were each identified in 1 isolate. The remaining 1 *exoU*+/*exoS*− isolate belonged to ST606 (no. 9). ST348 with an *exoU*−/*exoS*+ virulotype was detected in 2 isolates, namely, no. 10 and 16 (11.8%), of which 1 exhibited high-level resistance to imipenem (MIC, 128 mg/liter).

### Genomic features of carbapenem-resistant isolates.

Three representative P. aeruginosa isolates, including 2 showing high-level resistance to imipenem (no. 2 and 10; MICs, ≥ 64 mg/liter) and 1 isolate of the ST235 international high-risk clone (no. 13), were subjected to whole-genome sequencing (WGS). The assembled genomes of isolate no. 2, 10, and 13 contained 71, 118, and 143 contig sequences, with a total length of 6,270,234 bp, 6,831,356 bp, and 6,646,878 bp, respectively, with a GC content ranging from 66.1% to 66.5%.

Multiple antimicrobial resistance genes, including species-intrinsic *bla*_OXA-50-like_, *bla*_PDC_, *aph(3′)-IIb*, *fosA*, and *catB7* were found among the 3 isolates, where ST1600/O3 (isolate no. 2) harbored newly identified *bla*_OXA-937_ (Table 5). In addition, ST348/O2 (isolate no. 10) with a ciprofloxacin MIC of 0.5 mg/liter harbored *crpP* (ciprofloxacin-modifying enzyme) that was recently identified in a P. aeruginosa clinical isolate in Mexico (21). ST235/O11 (isolate no. 13) also contained 2 different class 1 integron-associated gene cassettes, and 1 contained *qnrVC1*-*aac(6′)-Ib*-*bla*_OXA-10_-*aadA1*-*dfrA14* and the other contained *aadB*-*cmlA10*-*aadA2*-*sul1*, conferring resistance to quinolones, aminoglycosides, β-lactams, trimethoprim, chloramphenicol, and sulfonamide. Different amino acid substitutions in AmpR were observed in the ST348/O2 and ST235/O11 isolates. The ST1600/O3 isolate with a ciprofloxacin MIC of 32 mg/liter had Ser466Phe in the quinolone resistance-determining region (QRDR) of GyrB.

**TABLE 5:** Genetic characteristics of P. aeruginosa isolates no. 2, no. 10, and no. 13

Characteristic	Results for:		
	Isolate 2 (CA12133)	Isolate 10 (CA17343)	Isolate 13 (CA19603)
Sequence type/serotype	ST1600/O3	ST348/O2	ST235/O11
Antimicrobial resistance genes	*bla*_PDC-3_, *bla*_OXA-937_, *aph(3′)-IIb*, *fosA*, *catB7*	*bla*_OXA-494_, *bla*_PDC-5_, *aph(3′)-IIb*, *fosA*, *catB7*, *crpP*	*bla*_OXA-488_, *bla*_PDC-35_, *aph(3′)-IIb*, *fosA*, *catB7*, *qnrVC1*-*aac(6′)-Ib*-*bla*_OXA-10_-*aadA1*-*dfrA14*, *aadB*-*cmlA10*-*aadA2*-*sul1*
Amino acid substitutions^*a*^			
AmpC regulators			
AmpR		Arg244Trp, Gly273Glu	Gly283Glu, Met288Arg
AmpD	Ser5Phe, Gly148Ala, Asp183Tyr		Gly148Ala
AmpDh2			
AmpDh3	Ile67Thr	Ala219Thr	Ala208Val
MexAB-OprM regulators			
MexR			Val126Glu
NalC	Gly71Glu, Ser209Arg		Glu153Gln
NalD			
MexCD-OprJ regulator			
NfxB	Arg21His, Asp56Gly, Gly133Ser		
MexEF-OprN regulators			
MexS	Asp249Asn	Gly78Ser, Asp249Asn	frameshift (Δnt 362T)
MexT			
MexXY-OprA regulator			
MexZ	Frameshift (Δnt 399G)		
OprD mutations^*a*^	Thr103Ser, Lys115Thr, Phe170Leu	Premature stop codon (W65stop)	Δnt 1–75
QRDR mutation^*a*^	Ser466Phe (GyrB)		
Heavy metal resistance genes	*copRS*, *copAB*, *czcABCD*, *colRS*, *chrA*, *sodA*	*copRS*, *copAB*, *copD*, *czcABCD*, *colRS*, *chrA*, *sodA*	*copRS*, *copAB*, *czcABCD*, *colRS*, *chrA*, *sodA*
Virulence-associated genes	T1SS (*aprA*, *aprX*, *hasAp*), T2SS (*lasA*, *lasB*, *plcH*, *plcN*, *plcB*, *prpL*, *phoA*, *lipA*, *lipC*, *loxA*, *toxA*, *paAP*), T3SS (*exoT*, *exoS*, *exoY*), H2-T6SS (*tssB2*, *pldB*), H3-T6SS (*tssB3*), QS (*lasI*, *lasR*, *rhlI*, *rhlR*), others (*fliC*, *rhlAB*)	T1SS (*aprA*, *aprX*, *hasAp*), T2SS (*lasA*, *lasB*, *plcH*, *plcN*, *plcB*, *prpL*, *phoA*, *lipA*, *lipC*, *loxA*, *toxA*, *cbpD*, *paAP*), T3SS (*exoT*, *exoS*, *exoY*), H2-T6SS (*tssB2*, *pldB*), H3-T6SS (*tssB3*), QS (*lasI*, *lasR*, *rhlI*, *rhlR*), others (*fliC*, *rhlAB*)	T1SS (*aprA*, *aprX*, *hasAp*), T2SS (*lasA*, *lasB*, *plcH*, *plcN*, *plcB*, *prpL*, *phoA*, *lipA*, *lipC*, *loxA*, *toxA*, *cbpD*, *paAP*), T3SS (*exoT*, *exoU*, *exoY*), H2-T6SS (*tssB2*, *pldA*, *pldB*), H3-T6SS (*tssB3*), QS (*lasI*, *lasR*, *rhlI*, *rhlR*), others (*fliC*, *rhlAB*)

a Amino acid substitution compared to the sequences of Pseudomonas aeruginosa strain PAO1 (GenBank accession number AE004091).

A mutated OprD porin by amino acid substitutions Thr103Ser, Lys115Thr, and Phe170Leu (ST1600/O3); a premature stop codon (ST348/O2); and a 75-bp deletion leading to a frameshift (ST235/O11) were identified (Table 5). These isolates had amino acid substitutions in the MexAB-OprM repressors MexR and NalC, Gly71Glu and Ser209Arg in NalC (ST1600/O3), and Val126Glu in MexR and Glu153Gln in NalC (ST235/O11). The substitutions of Arg21His, Asp56Gly, and Gly133Ser in NfxB of MexCD-OprJ and Asp249Asn in MexS of MexEF-OprN were found in the ST1600/O3 isolate. Gly78Ser and Asp249Asn in MexS of MexEF-OprN were also noted in the ST348/O2 isolate. In the ST235/O11 isolate, a frameshift mutation (a deletion of a T at position 362) was identified in MexS.

Three isolates shared virulence genes encoding secretion system components of types I (T1SS) and II (T2SS), LasR-LasI and RhlR-RhlI quorum-sensing (QS) systems, FliC flagellin, and rhamnolipid synthetic enzyme RhlAB (22, 23). However, different types of gene clusters were found in T3SS, namely, *exoT*/*exoS*/*exoY* for ST1600/O3 and ST348/O2 isolates and *exoT*/*exoU*/*exoY* for an ST235/O11 isolate. Also, three isolates shared *pldB* (H2-T6SS effector encoding gene), whereas only one ST235/O11 isolate also carried *pldA* (H2-T6SS effector encoding gene) (24). Heavy metal resistance genes, including those associated with copper (*copRS*, *copAB*, and *copD*), cobalt/zinc/cadmium (*czcABCD*), chromate (*chrA*), cadmium (*sodA*), and *colRS* regulating multimetal resistance, were identified among them (25).

### Whole-genome multilocus sequence typing (wgMLST).

**FIG 1:**
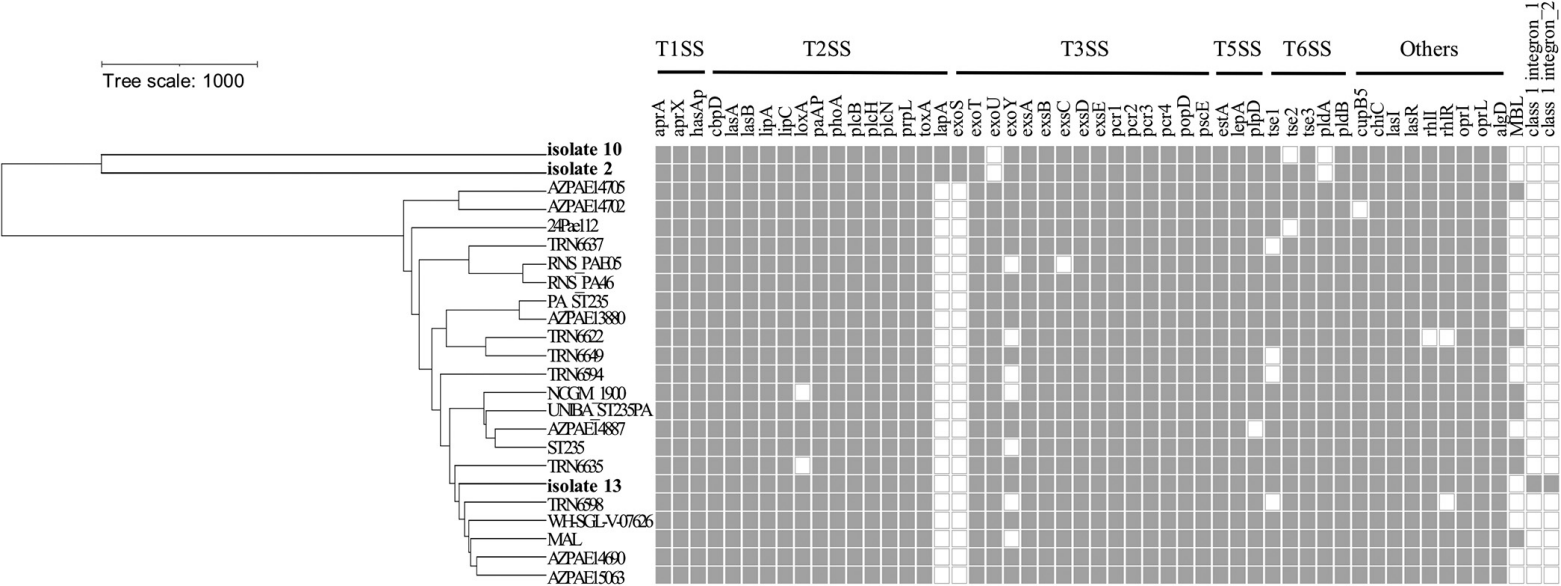
Whole-genome multilocus sequence typing (wgMLST) of P. aeruginosa ST235/O11 isolates. The carbapenem-resistant, carbapenemase nonproducing ST235/O11 isolate 13 (CA19603) in this study and 21 representative strains of human origin were analyzed. The presence (gray) or absence (white) of virulence-associated genes, metallo-β-lactamase (MBL) genes, and class 1 integron-associated gene cassettes among strains are shown. ST1600/O3 (isolate 2, CA12133) and ST348/O2 (isolate 10, CA17343) in this study are also included for comparison. MBL, *bla*_IMP_, *bla*_NDM_, and *bla*_VIM_; class 1 integron_1, *qnrVC1*-*aac(6′)-Ib*-*bla*_OXA-10_-*aadA1*-*dfrA14*; class 1 integron_2, *aadB*-*cmlA10*-*aadA2*-*sul1*.

The genomic diversity of the ST235 isolates was assessed by wgMLST gene-by-gene comparison. The animal-derived ST235/O11 isolate with *exoU*+/*exoS*− virulotype (isolate 13, CA19603) clustered with 21 ST235 isolates of human origin characterized by the *exoU*+ genotype (Fig. 1). They shared mostly important virulence-associated genes, including *pldA* and *pldB*. It is worth noting that the class I integron-associated gene cassettes that confer antimicrobial resistance, i.e., *qnrVC1*-*aac(6′)-Ib*-*bla*_OXA-10_-*aadA1*-*dfrA14* and *aadB*-*cmlA10*-*aadA2*-*sul1*, were identified in isolate 13.

## DISCUSSION

The perspective of One Health emphasizes the importance of AMR monitoring and integrated actions across human, food, animal, and environmental health. However, there are few studies on the epidemiology of antimicrobial resistance in P. aeruginosa clinical isolates from companion animals that share a living environment with humans. The strength of our study is that we included 240 P. aeruginosa clinical isolates collected from 152 primary care animal hospitals across the country, allowing us to understand the current situation of their antimicrobial resistance in companion animals as well as to investigate the resistance trends over time in Japan by comparing the results with those in earlier studies (19, 20). Namely, a trend toward a progressive increase in the frequency of imipenem resistance, from 0% in 2003 to 2010 (19), to 0.5% in 2014 to 2015 (20), to 6.67% in 2017 to 2019 (this study) is noted according to the breakpoints of the Clinical and Laboratory Standards Institute (CLSI) M100 30th edition guidelines (26).

The carbapenem resistance rates of isolates from companion animals were found to be similar to those from human outpatients in Japan, leading to concerns about the transmission of carbapenem-resistant P. aeruginosa between humans and companion animals in community settings. The ciprofloxacin resistance rate of 17.92% in our study is nearly flat from the previous years; 23.39% was found in 2003 to 2010 and 14.87% in 2014 to 2015 (19, 20). Constant amounts of veterinary fluoroquinolones such as enrofloxacin and orbifloxacin, at 0.90 tons per year during 2013 and 2018, have been used for dogs and cats as described in the Nippon AMR One Health Report (NAOR) 2020 (in Japanese, https://www.mhlw.go.jp/content/10900000/000715546.pdf). However, consumption of fluoroquinolones which are approved for human use such as levofloxacin and ciprofloxacin in companion animals cannot be traced because their prescription is left to the discretion of veterinarians in Japan. Thus, attention is necessary to monitor for high levels of resistance to ciprofloxacin, which is an active metabolite of enrofloxacin in companion animals, because ciprofloxacin is an effective treatment option in many human diseases. In contrast, piperacillin, piperacillin-tazobactam, ceftazidime, and cefepime retained activity against >99% of animal isolates, which is greater than the activity against isolates from hospital inpatients and outpatients (Table 1). Interestingly, in companion animals, none of the carbapenem-resistant isolates produced carbapenemases, and no multidrug-resistant P. aeruginosa isolates (MDRP) as defined by the Infectious Diseases Control Law in Japan (resistant to imipenem [MIC, ≥16 mg/liter], amikacin [MIC, ≥32 mg/liter], and ciprofloxacin [MIC, ≥4 mg/liter]) were identified, which is consistent with observations in previous animal studies (19, 20). However, our study detected four pre-MDRP isolates with imipenem MICs of ≥16 mg/liter and ciprofloxacin MICs of ≥4 mg/liter, emphasizing the need for continuous surveillance of resistance trends of this important pathogen.

To our knowledge, only one study has analyzed the T3SS virulotypes in a limited number of P. aeruginosa isolates from canine, reporting that 3 of 19 isolates from ocular infections were *exoU*+ genotypes (18). This study revealed that the prevalence of T3SS virulence genes among 240 clinical isolates of canine and feline origin (15.8% for *exoU* and 82.5% for *exoS*) is more similar to that of 90 environmental isolates (17.8% for *exoU* and 82.2% for *exoS*) than that of 243 human isolates from bloodstream infections (20.6% for *exoU* and 76.1% for *exoS*) (12, 27). There was no significant association between the *exoU*+/*exoS*− virulotype and ciprofloxacin and carbapenem resistance, contrary to some other studies reporting such an association in human isolates (11, 28). However, the *exoU*+ genotype has been an independent risk factor for early mortality of human bloodstream infections (11, 12), and our results draw attention to the prevalence of *exoU*+ isolates in a greater number of animal isolates.

Among global high-risk clones with MDR/XDR profiles, ST235 is highly associated with *exoU*, and this combination has been a predictor of a highly unfavorable prognosis (29). Thus, MLST analysis of *exoU*+ isolates is of clinical importance, as ST235 has not been confirmed among P. aeruginosa isolates from companion animals. MLST revealed clonal heterogeneity in the 38 *exoU*+ isolates, including *exoU*+/*exoS*− and *exoU*+/*exoS*+ virulotypes, with the presence of 20 STs, including 3 newly identified STs. Notably, the global high-risk clone ST235 was detected in 7 *exoU*+/*exoS*− isolates and in 1 *exoU*+/*exoS*+ isolate. Moreover, 3 CC235 isolates were found in 2 *exoU*+/*exoS*− isolates and in 1 *exoU*+/*exoS*+ isolate. Thus, 11 ST235/CC235 isolates were detected in 38 *exoU*+ isolates (28.9%), resulting in an overall ST235/CC235 frequency of 20.8% in 53 isolates with the *exoU*+ genotype and/or carbapenem resistance that were typed by MLST. In contrast, ST235/CC235 isolates were not found in 15 *exoU*+/*exoS*− isolates resistant to carbapenems. The CC235 has been linked with metallo-β-lactamase genes and, hence, is responsible for their dissemination worldwide, including to Japan (4, 30). Our findings provide important insights into the role of companion animals as the potential reservoirs of P. aeruginosa high-risk clones, although they did not harbor metallo-β-lactamase genes.

In P. aeruginosa, mutational overexpression of a multidrug efflux pump, MexAB-OprM, leads to reduced susceptibility to fluoroquinolones and most β-lactams, including meropenem, while imipenem is not affected (31). MexAB-OprM overexpression combined with OprD inactivation produces high-level meropenem resistance (MIC, >32 mg/liter) (32). Mutational inactivation of OprD contributes mainly to imipenem resistance, even achieving high-level resistance (MIC, >32 mg/liter), but drives only moderate resistance to meropenem (32). In addition, OprD inactivation, in combination with AmpC β-lactamase overexpression, confers resistance to carbapenems (33). In the present study, 7.1% (17/240) of isolates from companion animals were found to be carbapenem resistant but carbapenemase non-IMP producers. MIC assays performed in the presence of an efflux pump inhibitor PAβN and/or AmpC β-lactamase inhibitor APB revealed the contribution of AmpC β-lactamase production to imipenem resistance in 10 of 12 imipenem-resistant meropenem-susceptible isolates. In the remaining 2 isolates, the active role of efflux pumps (isolate 8) or the synergistic role of AmpC β-lactamase production and efflux pumps (isolate 2, imipenem MIC of 64 μg/ml) in mediating imipenem resistance is estimated. Among 4 imipenem- and meropenem-resistant isolates, AmpC β-lactamase-mediated imipenem resistance is considered in 3 isolates, including 1 also exhibiting efflux pump-mediated meropenem resistance (isolate 13, imipenem MIC of 32 mg/liter, meropenem MIC of 8 mg/liter). The involvement of the synergistic effect of AmpC β-lactamase production and efflux pumps in imipenem resistance and that of the efflux pump mechanism in meropenem resistance could be considered in the remaining 1 isolate (isolate 10, imipenem MIC of 128 mg/ml, meropenem MIC of 16 mg/liter). Efflux pump-mediated meropenem resistance is suggested for a meropenem-resistant imipenem-susceptible isolate.

Representative isolates analyzed by WGS contained defective *oprD* mutations in ST348/O2 (isolate 10) and ST235/O11 (isolate 13), while 3 amino acid substitutions observed in ST1600/O3 (isolate 2) may not be involved in carbapenem resistance because they have been frequently detected among carbapenem-susceptible isolates, including P. aeruginosa PA14 (34). Also, substitutions in regulators of AmpC β-lactamases (PDC variants) are found in these isolates, i.e., AmpR (Gly283Glu and Met288Arg), AmpD (Gly148Ala and Asp183Tyr), and AmpDh3 (Ala208Val and Ala219Thr), which have frequently been identified in wild-type isolates and P. aeruginosa PA14, and are considered nonsignificant (35, 36). All substitutions in MexAB-OprM regulators, namely, Arg21His and Asp56Gly in NfxB repressor of MexCD-OprJ and Asp249Asn in MexS repressor of the MexEF-OprN, have been found in susceptible isolates, including P. aeruginosa PA14, and thus may not be related to the overexpression of those efflux pumps (37–39). In consequence, carbapenem resistance may arise from AmpC derepression due to Ser5Phe in AmpD and/or Ile67Thr in AmpDh3 (40) combined with MexCD-OprJ overexpression due to Gly133Ser in NfxB in the ST1600/O3 isolate. A significant association between MexCD-OprJ overexpression and meropenem and imipenem resistance has been described (41). Carbapenem resistance in ST348/O2 and ST235/O11 isolates may be attributed to mutational inactivation of OprD combined with MexEF-OprN overexpression due to Gly78Ser or a frameshift mutation in MexS (39). MexEF-OprN overexpression, with a concomitant loss of OprD caused by mutated MexS, has been linked to carbapenem resistance (42). Besides, the involvement of AmpC (PDC) variants in decreased imipenem susceptibility cannot be excluded (43).

In conclusion, this study confirms the presence of high-risk clones in canine and feline isolates with the *exoU*+ genotype; ST235/CC235 isolates were the most predominant, followed by ST357 isolates. These animal isolates belonging to high-risk clones were found to be carbapenemase nonproducers, although international high-risk clones have been associated frequently with carbapenemase production. Nonetheless, the predominant occurrence of ST235 among companion animals may represent a threat to public health because of the ability of this clone to acquire and spread resistance elements, including carbapenemase genes. A comprehensive survey of P. aeruginosa needs to be conducted to better understand the spread of antimicrobial resistance elements, STs, and T3SS virulotypes at the human-animal-environment interface and to assess their clinical implications on humans and animals.

## MATERIALS AND METHODS

### Bacterial strains.

A total of 240 nonduplicate clinical isolates of P. aeruginosa from dogs (*n *= 206) and cats (*n *= 34) were collected from 152 primary care animal hospitals across Japan between August 2017 and October 2019. Those isolates were received from different geographic regions, as follows: Kanto (*n *= 117 from 71 hospitals), Chubu (*n *= 32 from 25 hospitals), Kinki (*n *= 24 from 19 hospitals), Chugoku (*n *= 21 from 11 hospitals), Tohoku (*n *= 19 from 9 hospitals), Kyushu (*n *= 13 from 10 hospitals), Hokkaido (*n *= 9 from 4 hospitals), and Shikoku (*n *= 5 from 3 hospitals). Clinical sources of the isolates were ear (*n *= 149), urine (*n *= 33), skin (*n *= 28), nasal cavity (*n *= 16), uterus (*n* = 3), cornea (*n* = 2), and others (*n *= 9, including 3 isolates of unknown origin). P. aeruginosa was confirmed with matrix-assisted laser desorption ionization–time of flight mass spectrometry (MALDI-TOF MS) (Bruker Daltonics Japan, Yokohama, Japan) using ≥2.000 score cutoffs for species-level identification.

### Antimicrobial susceptibility testing.

MICs were determined by the broth microdilution method recommended by the CLSI using a custom-designed microtiter panel (Kyokuto Optopanel MP; Kyokuto Pharmaceutical, Tokyo, Japan), and the results were interpreted according to CLSI M100 30th edition guidelines for human (26). Escherichia coli ATCC 25922 and P. aeruginosa ATCC 27853 were used as quality-control strains.

### Detection of T3SS virulence genes (*exoU* and *exoS*).

All isolates were subjected to PCR detection of T3SS effector toxin genes *exoU a*nd *exoS* using specific primers as shown in Table 6 (44, 45). The amplification products from some arbitrarily selected isolates were sequenced to confirm the reliability of the results.

**TABLE 6:** Primers used for PCR amplification in this study

Gene by type	Primer sequence^*a*^ (5′ to 3′)	Amplicon size (bp)
T3SS virulence genes		
* exoU*	F; ATGCATATCCAATCGTTG	2,000
	R; TCATGTGAACTCCTTATT	
* exoS*	F; CTTGAAGGGACTCGACAAGG	504
	R; TTCAGGTCCGCGTAGTGAAT	
Carbapenemase genes		
* bla*_IMP_	F; ACCGCAGCAGAGTCTTTGCC	587
	R; ACAACCAGTTTTGCCTTACC	
* bla*_NDM_	F; GGGCCGTATGAGTGA	758
	R; GAAGCTGAGCACCGCATTAG	
* bla*_VIM_	F; ATGTTCAAACTTTTGAGTAAG	801
	R; CTACTCAACGACTGAGCG	
* bla*_GES_	F; ATGCGCTTCATTCACGCAC	864
	R; CTATTTGTCCGTGCTCAGGA	
MLST		
* acsA*	F; ACCTGGTGTACGCCTCGCTGAC	842
	R; GACATAGATGCCCTGCCCCTTGAT	
* aroE*	F; TGGGGCTATGACTGGAAACC	825
	R; TAACCCGGTTTTGTGATTCCTACA	
* guaA*	F; CGGCCTCGACGTGTGGATGA	940
	R; GAACGCCTGGCTGGTCTTGTGGTA	
* nuoD*	F; ACCGCCACCCGTACTG	1,042
	R; TCTCGCCCATCTTGACCA	
* ppsA*	F; GGTCGCTCGGTCAAGGTAGTGG	989
	R; GGGTTCTCTTCTTCCGGCTCGTAG	
* trpE*	F; GCGGCCCAGGGTCGTGAG	811
	R; CCCGGCGCTTGTTGATGGTT	
* mutL*	F; AGGTTCGCGACCTGTTCTT	688
	R; GGACTCTCCAGCACGCTCT	

a F, forward primer; R, reverse primer.

### Analysis of carbapenem-resistant P. aeruginosa isolates.

Detection of carbapenemase activity in P. aeruginosa isolates exhibiting resistance to imipenem and/or meropenem was performed using the modified carbapenem inactivation method CIMTris (46). Carbapenemase genes (*bla*_IMP_, *bla*_NDM_, *bla*_VIM_, and *bla*_GES_) were detected by PCR assay using specific primers as shown in Table 6.

The imipenem and meropenem MICs were determined using microdilution panels prepared in-house in the absence and in the presence of 20- or 40-mg/liter efflux pump inhibitor PaβN (MP Biomedicals, Santa Ana, CA), 20- or 40-mg/liter PAβN plus 300-mg/liter APB (Sigma-Aldrich Japan, Tokyo, Japan), and 300-mg/liter APB in parallel. P. aeruginosa ATCC 27853 was used as the control strain. MIC reduction (4-fold or greater) in the presence of PaβN and/or APB was considered the participation of efflux pump and/or AmpC β-lactamase in the resistance to those carbapenems.

### MLST analysis.

MLST for carbapenem-resistant isolates and *exoU*+ isolates was performed according to the MLST scheme described by Curran et al. except that Tks Gflex DNA polymerase (TaKaRa Bio, Otsu, Japan) and alternative PCR primers for *mutL* shown in Table 6 were used (47, 48). Briefly, seven housekeeping genes (*acsA*, *aroE*, *guaA*, *mutL*, *nuoD*, *ppsA*, and *trpE*) were amplified and sequenced. Those sequences were submitted to the PubMLST database (https://pubmlst.org/organisms/pseudomonas-aeruginosa) for the assignment of allelic numbers and STs. Those STs were assigned to clonal complexes, as defined by closely related ST groups within the single-locus variant or double-locus variant linkages (49).

### WGS analysis of high-level imipenem-resistant P. aeruginosa.

WGS and *de novo* assembly of three representative carbapenem-resistant isolates were conducted in a manner like that described previously (50). Briefly, the genome was sequenced using the 150-bp paired-end method with the NovaSeq6000 platform (Illumina, San Diego, CA), and the resulting raw reads were assembled *de novo* into scaffolds using the SPAdes v.3.14.1. The assembled scaffolds were queried with ResFinder 4.1 and PAst 1.0 that are available from the Center for Genomic Epidemiology (http://www.genomicepidemiology.org) for the prediction of antibiotic resistance genes and *in silico* serotyping, respectively. A wgMLST tree was constructed using PGAdb-builder (http://wgmlstdb.imst.nsysu.edu.tw), with the addition of the genomes of 21 P. aeruginosa strains belonging to ST235-O11 obtained from the NCBI database (Table 7). The phylogenetic tree was visualized using iTOL v5 (http://itol.embl.de/). An in-depth exploration of heavy metal resistance and virulence genes was performed manually on WGS data.

**TABLE 7:** List of 21 ST235/O11 P. aeruginosa reference genomes included in the wgMLST

Strain	Isolation source	Country	GenBank assembly accession no.
ST235	Homo sapiens	Italy	GCA_011634585.1
UNIBA_ST235PA	Blood	Italy	GCA_013276295.1
TRN6637	Homo sapiens	Russia	GCA_001921165.1
PA_ST235	Blood	Spain	GCA_000737795.1
MAL	Homo sapiens	France	GCA_901035125.1
24Pae112	Blood	Colombia	GCA_003433235.1
WH-SGI-V-07626	Homo sapiens	USA	GCA_001452025.1
RNS_PA46	Burn wound	Australia	GCA_001623955.1
RNS_PAE05	Hand sanitizer	Australia	GCA_001623985.1
TRN6594	Homo sapiens	Argentina	GCA_001921015.1
TRN6598	Homo sapiens	South Africa	GCA_001921025.1
TRN6622	Homo sapiens	Thailand	GCA_001921095.1
TRN6635	Homo sapiens	Russia	GCA_001921125.1
TRN6649	Homo sapiens	Nigeria	GCA_001921175.1
NCGM 1900	Homo sapiens	Japan	GCA_000829275.1
AZPAE13880	Homo sapiens	Mexico	GCA_000795725.1
AZPAE14887	Intra-abdominal tract infection	Croatia	GCA_000790725.1
AZPAE14690	Urinary tract infection	Romania	GCA_000794555.1
AZPAE14702	Respiratory tract infection	Philippines	GCA_000794785.1
AZPAE14705	Urinary tract infection	Greece	GCA_000795805.1
AZPAE15063	Respiratory tract infection	Brazil	GCA_000793315.1

The nucleotide sequences of AmpC β-lactamase regulator genes (*ampR*, *ampD*, *ampDh2*, and *ampDh3*); *oprD* porin genes; efflux system regulator genes (*mexR*, *nalC*, *nalD*, *mexZ*, *nfxB*, *mexS*, and *mexT*); and QRDR of *gyrA*, *gyrB*, *parC*, and *parE* genes were compared with those of the reference strain P. aeruginosa PAO1 (GenBank accession number AE004091).

### Analysis of ciprofloxacin-resistant P. aeruginosa isolates.

Efflux pump-mediated fluoroquinolone resistance was investigated in ciprofloxacin-resistant isolates using efflux pump inhibitor PAβN as described above. A decrease of more than 4-fold in MIC in the presence of PAβN was considered the participation of efflux pumps in the resistance to ciprofloxacin.

### Statistical analysis.

The Fisher’s exact test was performed to assess the association between T3SS virulotypes (*exoU*+/*exoS*− and *exoU*−*exoS*+) and antimicrobial resistance by using R v4.0.5. Resistant and intermediate isolates were grouped as resistant for statistical analyses. *P* value of *≤*0.05 were considered statistically significant.

### Data availability.

Genome assemblies for P. aeruginosa isolates no. 2 (CA12133), 10 (CA17343), and 13 (CA19603) have been deposited in the DDBJ/EMBL/GenBank database under the GenBank assembly accession numbers GCA_015704805.1, GCA_014596255.1, and GCA_014596275.1, and the BioProject accession number PRJNA648803.
